# Genome-wide identification of the NAC family in *Hemerocallis citrina* and functional analysis of *HcNAC35* in response to abiotic stress in watermelon

**DOI:** 10.3389/fpls.2024.1474589

**Published:** 2024-10-14

**Authors:** Lihong Cao, Jinyao Wang, Sijia Ren, Yumei Jia, Yue Liu, Shanjie Yang, Junshen Yu, Xinjuan Guo, Xiaojie Hou, Jin Xu, Sen Li, Guoming Xing

**Affiliations:** Shanxi Key Laboratory of Germplasm Resources Innovation and Utilization of Vegetable and Flower, College of Horticulture, Shanxi Agricultural University, Taigu, China

**Keywords:** *Hemerocallis citrina*, NAC transcription factor, genome-wide identification, abiotic stress, expression analysis

## Abstract

**Introduction:**

NAC (NAM, ATAF, and CUC) transcription factor family, one of the important switches of transcription networks in plants, functions in plant growth, development, and stress resistance. Night lily (*Hemerocallis citrina*) is an important horticultural perennial monocot plant that has edible, medicinal, and ornamental values. However, the *NAC* gene family of night lily has not yet been analyzed systematically to date.

**Methods:**

Therefore, we conducted a genome-wide study of the HcNAC gene family and identified a total of 113 HcNAC members from the Hemerocallis citrina genome.

**Results:**

We found that 113 HcNAC genes were unevenly distributed on 11 chromosomes. Phylogenetic analysis showed that they could be categorized into 16 instinct subgroups. Proteins clustering together exhibited similar conserved motifs and intron–exon structures. Collinearity analysis indicated that segmental and tandem duplication might contribute to the great expansion of the *NAC* gene family in night lily, whose relationship was closer with rice than *Arabidopsis*. Additionally, tissue-specific pattern analysis indicated that most *HcNAC* genes had relatively higher expression abundances in roots. RNA-Seq along with RT-qPCR results jointly showed *HcNAC* genes expressed differently under drought and salinity stresses. Interestingly, *HcNAC35* was overexpressed in watermelon, and the stress resilience of transgenic lines was much higher than that of wild-type watermelon, which revealed its wide participation in abiotic stress response.

**Conclusion:**

In conclusion, our findings provide a new prospect for investigating the biological roles of *NAC* genes in night lily.

## 1 Introduction

Transcription factors (TFs), known as master regulators of gene expression, bind to the specific responsive elements within the promoters of target functional genes. A series of plant-specific TFs, such as bHLH, bZIP, ARF, DREB, MYC, AP2/EREBP, WRKY, and NAC, regulate many biological processes (Deng et al., 2016; Droge-Laser et al., 2018; Erpen et al., 2018; Ohta et al., 2018; Li et al., 2019; Luo et al., 2020; Yao et al., 2020). The *NAC* gene family, one of the largest TF superfamilies of TFs in plants, was named from NAM (petunia no apical meristem), AF1/2 (*A. thaliana* transcriptional activator 1/2) and cup-shaped cotyledon (Han et al., 2023). The *NAC* gene family was widely characterized in dicotyledonous and monocotyledonous based on the availability of complete plant genome sequences. Among these plant species, 117 in *Arabidopsis* (Ooka et al., 2003), 151 in rice (Nuruzzaman et al., 2010), 152 in soybean (Le et al., 2011), 251 in switchgrass (Yan et al., 2017), 72 in perennial ryegrass (Nie et al., 2020), 93 in tomato (Jin et al., 2020), 180 in apple (Su et al., 2013), 170 in poplar (Meng L. et al., 2022), 74 in grape (Wang N. et al., 2013), 183 genes in white pear (Gong et al., 2019), and 154 in tobacco (Li et al., 2018) were investigated.

Typically, NAC protein mainly contained a highly conserved DNA-binding domain (BD) at the N terminus and a diverse C-terminal transcriptional activation region (TR) (Ooka et al., 2003; Puranik et al., 2012). Therefore, the NAC TFs are involved in various processes including growth, development, and stress responses (Puranik et al., 2012). The crucial role of NAC TFs in secondary cell wall development is discovered through establishing gene regulatory networks in *Arabidopsis* (Taylor-Teeples et al., 2015). ONAC127 and ONAC129 function indispensably in seed germination by directly targeting *OsMST6* and *OsSWEET4* (Ren et al., 2021). AtNAC1 coordinates with the SCR/SHR-CYCD6 to regulate the maturation of the root ground tissue in *Arabidopsis* (Xie et al., 2023). AtNAC056 upregulates the expression of *NIA1* by directly binding to its promoter, promoting root growth (Xu et al., 2022). The NAC family members ZmNAC126, BnaNAC60, MdNAC4, and LpNAL were involved in leaf senescence by positively regulating the expression of *SAGs* and *CGGs* (Yang et al., 2020; Yan et al., 2021; Wen et al., 2022; Yu et al., 2022). AdNAC2 and AdNAC72 indirectly regulate the ethylene pathway by respectively regulating the promoter and transcript of *AdMsrB1* (Fu et al., 2021). NAC68 positively regulates sugar accumulation and IAA levels and improves fruit quality and seed development in watermelon (Wang et al., 2021).

As the main environmental stress elements, extreme temperatures, high salinity, drought, and other abiotic stresses have unfavorable effects on agricultural crop production either alone or in combination (Bashir et al., 2019). Several researches have suggested the implication of NAC TFs in response to abiotic stress in plants. For instance, NAC25 and NAC28 in bananas negatively regulated cold tolerance through phospholipid degradation-related pathways (Song C. B. et al., 2022). SlNAM3 in tomatoes enhances cold resistance (Dong et al., 2022). LlNAC014 senses high temperatures by binding directly to the promoter cis-element CTT(N7) AAG (Wu et al., 2022). ZmNAC074 positively regulates thermotolerance in maize (Xi et al., 2022). *RcNAC72* enhances drought tolerance by interacting with RcDREB1A in roses (Jia et al., 2022). Overexpression of *OsNAC2* improved drought resistance by inhibiting ROS accumulation (Li et al., 2022). *SlNAC10* could enhance salt tolerance when ectopically overexpressed in *Arabidopsis* (Du et al., 2022). Overexpression of *IbNAC3* in *Arabidopsis* can confer tolerance to salinity stress by integrating ABA-signaling pathway (Meng X.Q. et al., 2022). Therefore, the multiple functions of NAC in plants need to be continuously explored.

Chinese night lily (*Hemerocallis citrina* Baroni, 2*n* = 22), one of the most important horticultural perennial crops in northeastern China, has edible, medicinal, and ornamental purposes (Cao et al., 2024). Night lily is also named long yellow daylily, exhibiting widespread involvements in abiotic stress responses (Zhang et al., 2023). In recent years, the production and consumption of night lily have increased with its dried immature flower buds as a primary food source (Zuo et al., 2024). Therefore, it is essential to investigate stress tolerances to improve the yield of night lily products and byproducts. Draft genome sequences of night lily released in 2021 have provided researchers with vital resources for genome-wide analysis of multiple gene families with specific functions (Qing et al., 2021). However, no systematic analysis of the night lily NAC TFs was performed. In the current study, 113 *HcNAC* genes representing 16 subgroups were identified. A comprehensive analysis of chromosomal distributions, phylogenetic relationships, domain analysis, motif compositions, gene structures, *cis*-acting elements in promoters, gene duplications, and collinearity analysis was completed. We also analyzed the night lily *NACs* tissue-specific expression profiles by RNA sequencing (RNA-Seq) and further quantitative reverse transcription polymerase chain reaction (RT-qPCR) verification. In addition, both transcriptome data and qPCR results showed that some *HcNAC* genes in addition to *HcNAC35* might response to abiotic stress treatments. Moreover, we examined the function of *HcNAC35* gene through ectopically overexpression in watermelon given higher sensitivity of watermelon to abiotic stress compared with most other crops (Lv et al., 2016). Our results showed that overexpression of *HcNAC35* could enhance abiotic tolerances especially salinity stress, which unveiled crucial mechanisms of night lily NAC-mediated response to salinity stress. Overall, the study provided valuable information relevant and a theoretical foundation for the functional investigation of night lily NAC family members.

## 2 Materials and methods

### 2.1 Identification and sequence analysis of *HcNAC* genes

To identify *HcNAC* genes, we downloaded the files of *H. citrina* including the genome sequences, coding sequences (CDS), and protein sequences from NCBI (https://www.ncbi.nlm.nih.gov/assembly/GCA_017893485.1) (Qing et al., 2021). Hidden Markov model (HMM) files were constructed based on the NAM domain (PF02365) retrieved from the Pfam website (https://pfam.xfam.org/). The conserved NAM domain was utilized to search for HcNAC protein sequences by a program of HMMER3.0 (*E*-value ≤ 10^−5^). Then, the physicochemical property prediction of HcNAC protein sequences was detected using TBtools software (Chen et al., 2020). The secondary structure prediction was performed by the online website (https://npsa-prabi.ibcp.fr/NPSA/npsa_sopma.html). WoLF PSORT software online (https://wolfpsort.hgc.jp/) was used to predict the subcellular localization of HcNAC proteins.

### 2.2 Chromosomal location, phylogeny, and gene structure analysis of the NAC family genes in night lily

We obtained the genome annotation GFF3 file of the *NAC* genes in night lily from the NCBI database, which was used for mapping the *HcNAC* gene chromosomal positions using one small program (Gene Location Visualize from GTF/GFF) of TBtools. *A. thaliana*, *O. sativa*, and *C. lanatus* NAC proteins were obtained from the Arabidopsis Information Resource (http://www.Arabidopsis.org/), rice genome annotation (http://rice.plantbiology.msu.edu/), and Cucurbit Genomics Database (http://cucurbitgenomics.org/), respectively (Ooka et al., 2003; Nuruzzaman et al., 2010; Lv et al., 2016). Combined with identified HcNAC proteins, a phylogenetic tree was constructed using the MEGA11.0.13 integrated tool by the Neighbor-Joining method (Kumar et al., 2016). The tree nodes were evaluated by 5,000 bootstrap replicates. *HcNAC* gene structure was analyzed using the Visualize Gene Structure of TBtools applets.

### 2.3 Gene motif, conserved domain, genome synteny, and Ka/Ks analysis

Gene-conserved motif prediction was performed via the MEME tool (http://meme-suite.org/index.html) with default settings. NCBI conserved domain database was used to predict the conserved domains of the HcNACs. Genome synteny analysis was made as described previously (Sun et al., 2017). We used Advanced Circos and dual synteny plot of TBtools software to show homologous gene pairs. Nonsynonymous (Ka) and synonymous (Ks) rates among protein sequences were used to assess the DNA sequence evolution. To appraise the divergence of duplicated night lily *NAC* genes, the selective strength was estimated by calculating the Ka/Ks ratio between paralogous gene pairs using the Simple Ka/Ks Calculator Tool (NG) in TBtools. Ka/Ks larger than 1 indicates positive selection, Ka/Ks less than 1 indicates purifying selection, and Ka/Ks equal to 1 indicates neutral mutation (Zhang and Yu, 2006).

### 2.4 Functional enrichment and *cis*-acting element analysis

Gene functional enrichment analysis was performed to reveal the biological processes, cellular components, and molecular functions of the *HcNAC* genes using STRING (https://cn.string-db.org/). The results were visualized using microscopic letter website (http://www.bioinformatics.com.cn/). For the *cis*-acting element analysis, about 2,000 base pairs of promoter regions upstream from the initiation codon of *HcNAC* genes were extracted and then analyzed by the PlantCARE database (http://bioinformatics.psb.ugent.be/webtools/plantcare/html/) to hunt for promoter *cis*-elements (Lescot et al., 2002).

### 2.5 Plant materials and stress treatments

In this study, *H. citrina* was used as treated plant species and cultivated in the experimental base. We chose F1 hybrid population 116 as the treated material based on its moderate resistance to abiotic stresses, obtained by Dongzhuang Huanghua as the female parent and Chonglihua as the male parent. Scapes were taken as explants for tissue cultivation (Zuo et al., 2024). These obtained seedlings were cultured in a 28°C growing box with the 16 h light/8 h dark condition and treated with Hoagland solution respectively containing 20% PEG6000 and 250 mM NaCl after 4 weeks old (Cao et al., 2024). Roots were collected at different time points with the same interval and frozen at a lower temperature than −70°C immediately for later use.

### 2.6 Transcriptome sequencing analysis

To investigate the transcriptional dynamics of the *HcNAC* genes in different tissues, bud, tender leaf, mature leaf, tender scape, mature scape, tender root, and mature root were collected and then sent to BMK Biotechnology Company for transcriptome sequencing analysis (NCBI accession: PRJNA1154842). We also chose collected roots of five groups (0, 24, 48, 72, and 108 h) under drought stress (NCBI accession: PRJNA1154840) and five groups (0, 24, 48, 72, and 96 h) under salinity stress for RNA-Seq analysis (NCBI accession: PRJNA1154841). All samples were designed for three replicates. Raw data were performed quality control checks through FastQC and filtered by Trimmomatic 0.36 using the quality control results (Bolger et al., 2014). HISAT2 2.2.1 was used to map the paired-end clean reads to the *H. citrina* reference genome (Kim et al., 2015). DESeq2 1.30.1 were used to determine differentially expressed genes with the analysis of variance (ANOVA) method (*p*.adjust < 0.05, |Log_2_FC| ≥ 1.5) (Love et al., 2014). The fragments per kilobase per million mapped fragments (FPKM) values of *HcNAC* genes were summed and used for measuring the transcript abundance of *HcNACs*. The heat map was generated with log_2_FPKM values to visualize different expression levels.

### 2.7 Reverse-transcription quantitative real-time PCR analysis

RT-qPCR analysis was carried out according to previously reported with little modification (Hu et al., 2016). The cDNA was synthesized by reverse transcription of high-quality RNA using the TIANScript II RT Kit by the corresponding instructions (TianGen, Beijing, China). RT-qPCR–specific primers, designed using Primer Premier 5, were shown in **Supplementary Table S11**. The gene expression levels were detected using a LightCycler480 II (Roche, Basel, Switzerland). FastKing One-Step SYBR Green Kit (TianGen, Beijing, China) was used for RT-qPCR reactions, and the calculation of the gene expression levels was analyzed through the 2^−ΔΔCt^ Method. Significant difference in gene expression levels between the two treatment groups was analyzed by a one-way ANOVA analysis of variance method with Duncan test at a *p*-value < 0.05. Gene expression was analyzed using three independent biological repeats. To normalize expression levels of the selected *HcNAC* genes, *HcACTIN* gene was used as an internal control (Cao et al., 2024).

### 2.8 Construction of *HcNACs* (*HcNAC35* and *HcNAC71*) transient expression vector and subcellular localization

*HcNAC35* and *HcNAC71* fragments with *Kpn*I and *Xho*I restriction sites were obtained by conventional PCR. The plasmid pSuper1300 with Green Fluorescent Protein (GFP) was digested with these two enzymes to get the linear vector fragment, which was subsequently ligated with the target fragments using pEASY-Basic Seamless Cloning and Assembly Kit (TransGen, Beijing, China). Constructed fusion expression plasmids including pSuper1300-HcNAC35-GFP and pSuper1300-HcNAC71-GFP were transformed into tobacco leaf cells by the *Agrobacterium*-mediated transient transformation. After 48–60 h of dark culture, GFP fluorescence signals were captured using a laser fluorescence microscope (Zeiss LSM800). Relative primers were listed in **Supplementary Table S11**.

### 2.9 Overexpression vector construction and transformation of *HcNAC35* in watermelon

The full-length CDS with the *Bam*HI and *Kpn*I restriction sites of HcNAC35 were amplified for constructing an overexpression vector and then cloned into a 1305.4-with Green Fluorescent Protein (GFP) vector to carry out sequencing Tongchuan verification. The resulting construct 1305.4-HcNAC35-GFP was transformed into *Agrobacterium tumefaciens* strain EHA105 to perform genetic transformation using an optimized transformation system in watermelon Tongchuan (TC) (Cao et al., 2022). Transgenic plants were screened by GFP observation and PCR analysis. Wild-type (WT) TC and transgenic plants were transplanted and cultivated in the same growing conditions. Primers referred to in the experiment were given in **Supplementary Table S11**.

### 2.10 Measurements of some physiological indicators

The levels of MDA (malondialdehyde) were assessed using the thiobarbituric acid method as described in previous study (Hu et al., 2019). Approximately 0.2 g leaf samples of both salinity-stressed and unstressed plants were collected and ground with 2 ml of ice-cold 0.5% TCA (trichloroacetic acid). The homogenates were centrifuged at 7,000 rpm for 15 min at 4°C. A 1.5-ml volume of the supernatant and 1.5 ml of 8% TCA containing 0.5% thiobarbituric acid were mixed, and boiled for 10 min. The homogenate cooled to ambient temperature was centrifuged at 7,000 rpm for 15 min. Absorbance was measured at 450, 532, and 600 nm.

The level of O_2_^−^ was also determined as previously described (Hu et al., 2019). Leaf samples (0.3 g per sample) were ground with 2 ml of ice and precooled 50 mM phosphate buffer solution (PBS, pH 7.8). The homogenates were then centrifuged at 10,000 rpm for 20 min at 4°C. A 1 ml of the supernatant, 250 μL of hydrochloride hydroxylamine, and 750 μL of 65 mM PBS (pH 7.8) were mixed and incubated for 1 h at 25°C. Then, a 1 ml of the reaction mixture, 1 ml of 7 nM α-naphthylamine, and 1 ml of 17 nM paminobenzenesulfonic acid were mixed and incubated for 30 min at 25°C. Finally, the absorbance was measured at 530 nm. The O_2_^−^ content was calculated using a standard curve relating O_2_^−^ concentration to absorbance. H_2_O_2_ was assessed using Micro Hydrogen Peroxide (H_2_O_2_) Assay Kit (Solarbio, Beijing, China). Absorbance was recorded at 450, 532, and 600 nm using an Infinite M200 microplate reader (Tecan, Männedorf, Switzerland).

## 3 Results

### 3.1 Genome-wide identification, feature, and phylogenetic analysis of the NAC family in night lily

**Figure 1:**
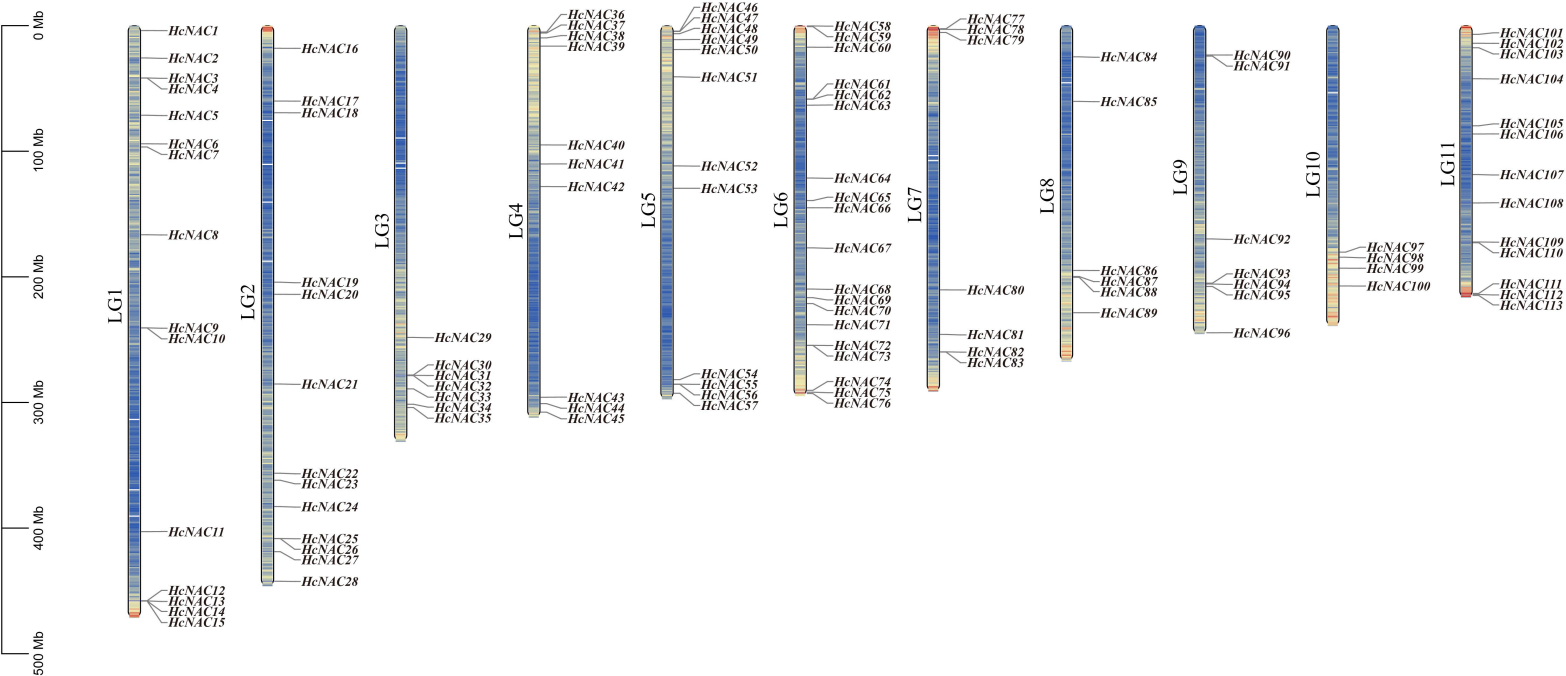
Locations of Hc*NAC* genes on chromosomes (LG1-11). The gene and chromosome names were labeled on the left of each strip. A chromosome length of 100 Mb was used as the basic unit. Lines and different colors inside the LGs indicated gene density differences.

Multiple sequence alignment analysis was performed, followed by a total of 113 complete non-redundant *NAC* genes identified based on the PlantTFDB database and the reported *H. citrina* genome (Qing et al., 2021). The chromosomal distributions of genes showed that they were unequally dispersed across LG1-LG11 with gene counts ranging from 4 to 19. These genes were designated *HcNAC1*-*HcNAC113* according to their chromosomal positions (**Figure 1**). The full length of encoded HcNAC proteins varied significantly from 94 to 1,285 amino acid residues. Physicochemical properties were further analyzed based on their protein sequences, with molecular weights ranging from 10.7 to 141.9 kDa, and theoretical pI values from 4.42 to 10.4 (**Supplementary Table S1**). The secondary structure including alpha helix, beta-turn, random coil, and extended strand was listed in **Supplementary Table S2**. The subcellular localization prediction indicated that most HcNAC proteins were localized in the nucleus region, but the others were mainly in either chloroplast or cytoplasm.

**Figure 2:**
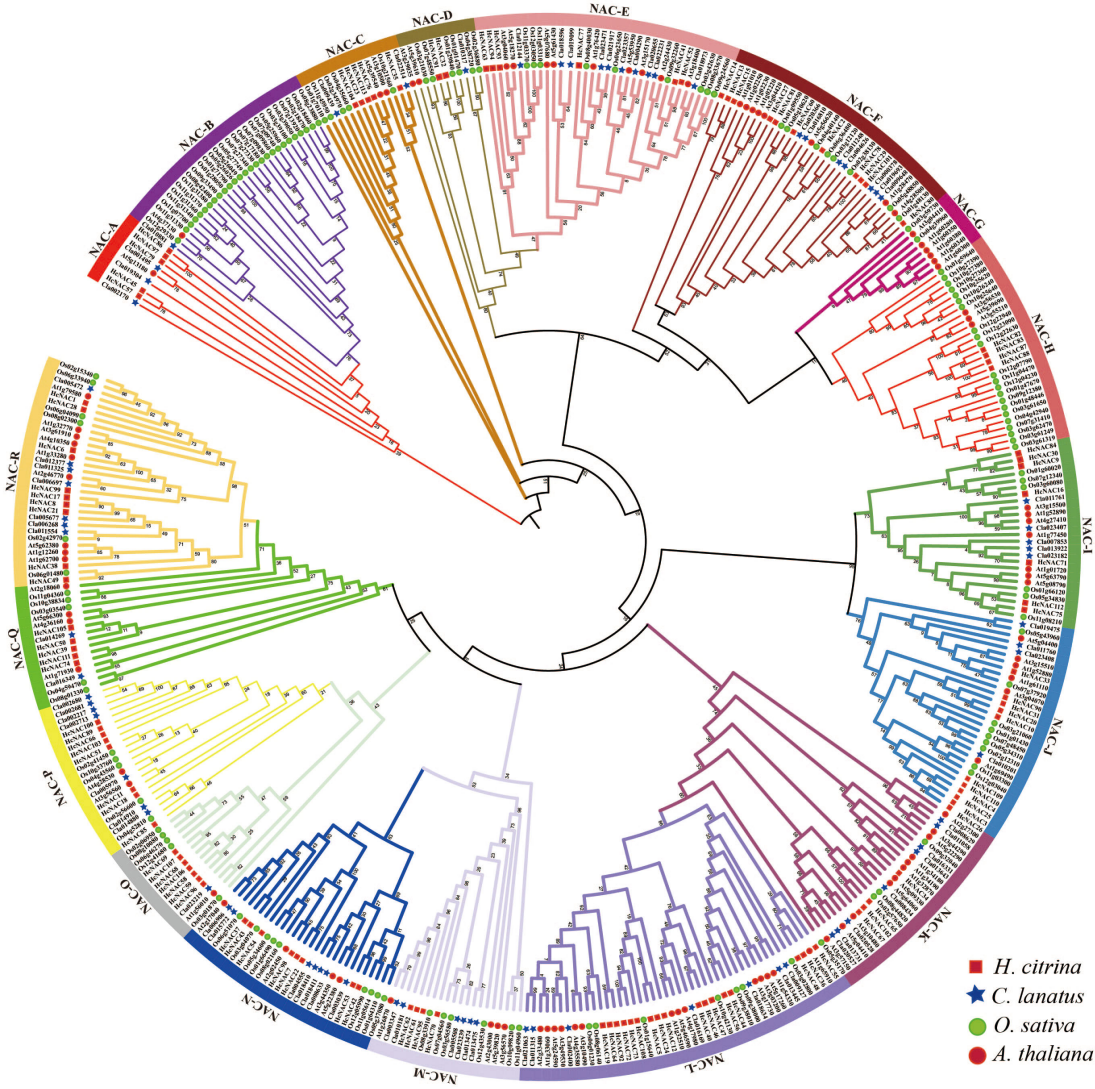
Phylogeny of the HcNACs in night lily, watermelon, and representative plants, including monocot *O. sativa* and eudicot *A. thaliana*. *NAC* genes of *H. citrina* (Hc), *C. lanatus* (Cla), *O. sativa* (Os), and *A. thaliana* (At), were clustered into 18 clades (A–R). *H. citrina* (red square), *C. lanatus* (blue pentagram), *O. sativa* (green circle), *A. thaliana* (red circle).

To explore the evolutionary relationships among *H. citrina* NAC TFs, a phylogenetic tree was established using the NAC protein sequences from *H. citrina, C lanatus*, *O. sativa*, and *A. thaliana* (**Figure 2**). The protein sequences (ClaNACs, AtNACs, OsNACs) involved in tree construction are listed in **Supplementary Table S3**. The phylogenetic analysis indicated that 113 HcNAC proteins tightly clustered with ClaNAC, AtNAC, and OsNAC proteins possessed 16 subgroups except the NAC-B and NAC-G subgroup, which suggested evolutionary conservation of the *HcNAC* gene family. The NAC-L subgroup comprised 17 *HcNACs*, 7 *ClaNACs*, 7 Os*NACs*, and 14 *AtNACs*, belonging to the divided largest subfamily. The NAC-D subgroup consisted of six Os*NAC* genes, but only two *HcNACs*, two *ClaNACs*, and two *AtNAC* genes. In the NAC-B subgroup, 27 *OsNACs*, 1 *ClaNAC*, and 1 *AtNAC* gene were found while no *NAC* gene was in *H. citrina*. The NAC-G subgroup contained six *AtNACs* and two *OsNAC* genes, but no *ClaNACs* and *HcNAC* genes. The types and number of genes within each subfamily varied greatly. HcNAC TF was absent in the NAC-B and NAC-G subgroups, which implies that these groups might be lost in night lily during evolution.

### 3.2 Domains, conserved motifs, and gene structural analysis of *HcNAC* genes

**Figure 3:**
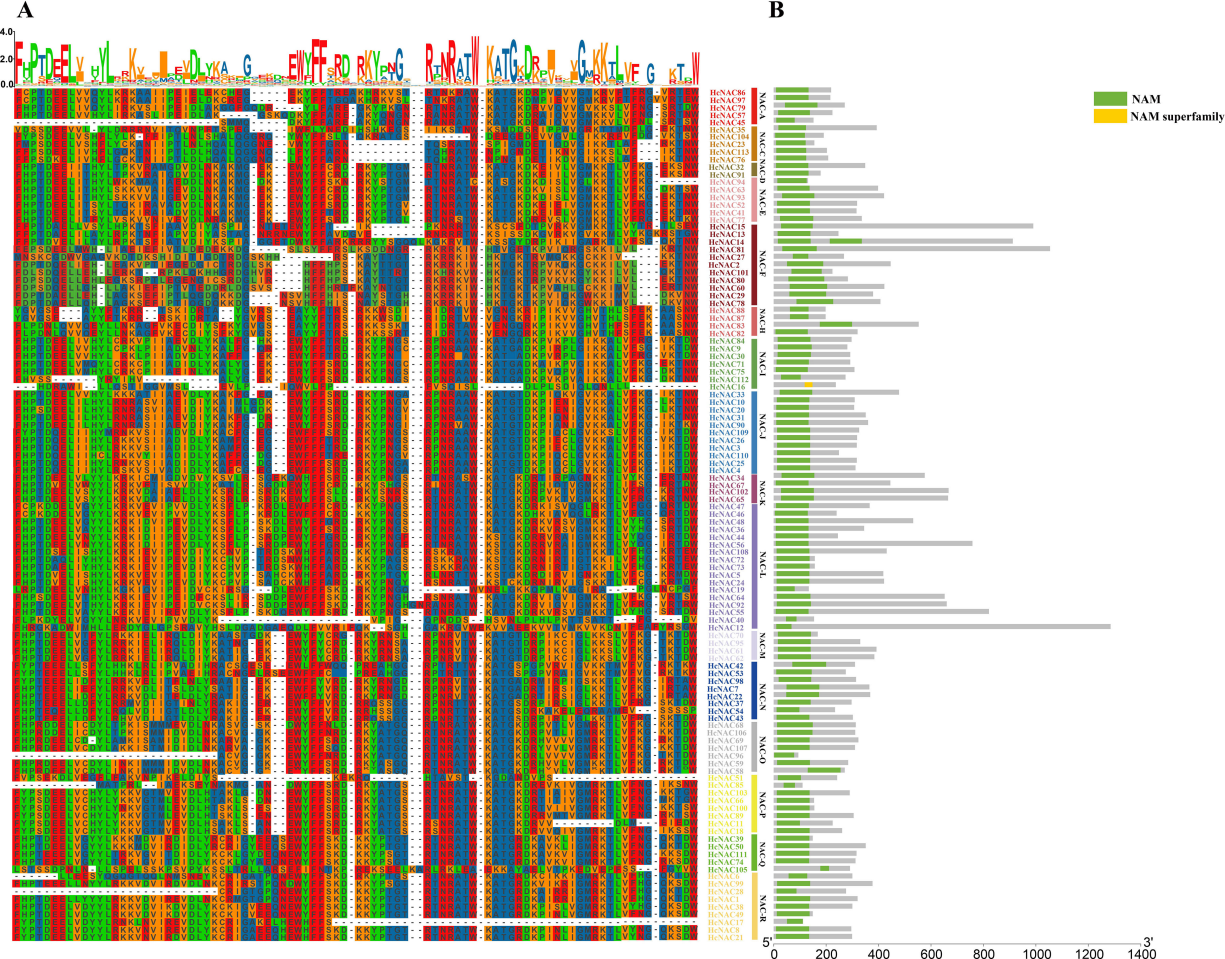
Multiple sequence alignment and conserved domains of 113 HcNAC proteins based on clustering results. **(A)** Protein sequence alignment in *H*. *citrina*. Incomplete or no N-terminal domain sequences were omitted from the alignment. **(B)** Distribution of conserved domains of 113 HcNAC proteins.

To further investigate the evolutionary conservation of *HcNAC* genes, multiple sequence alignment was conducted to explore the homologous domain features and frequency of amino acids, exhibiting high-sequence homology in the same subgroup and showing a bit of difference in the conservatisms of some amino acids among HcNAC proteins of different subgroups (**Figure 3A**). According to the above results of sequence alignment, we carried out conserved domain prediction (**Figure 3B**). NAM domain for DNA binding was detected in 112 NAC proteins. In addition, HcNAC16 had an incomplete subdomain named NAM superfamily. The results indicated that strong sequence conservation existed in their evolutionary process. The NAM domain was the core region for investigating the biological functions of NAC proteins in *H. citrina*.

**Figure 4:**
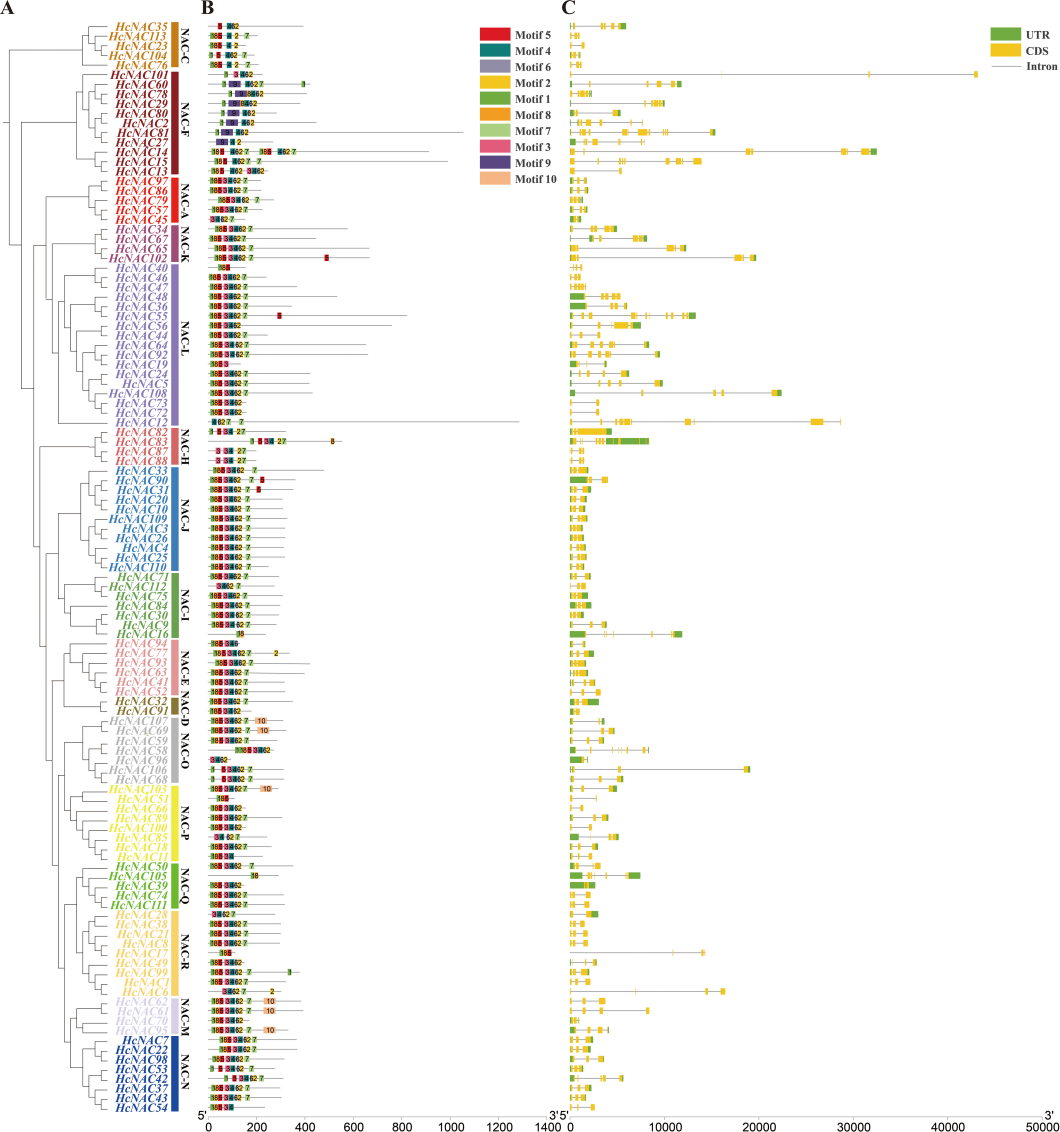
Phylogenetic tree, conserved protein motif, and gene structure analysis of *H*. *citrina* NAC family. **(A)** Clusters of the HcNAC proteins. The 16 subgroups were indicated (NAC-A, C–F, and H–R) and marked with different colors. **(B)** The distribution of putative conserved motifs of HcNAC proteins. **(C)** Structural variations in the genetic exon–intron regions, including untranslated regions (UTRs, green rectangle), CDSs (yellow rectangle), and introns (black line).

An analysis of gene motifs may help to better understand the diversity of the HcNAC proteins. The 113 HcNAC TFs were divided into 16 subgroups in the NJ phylogenetic tree. Among them, the NAC-L subgroup was the highest in numbers and NAC-D was the lowest with only two members (**Figure 4A**), consistent with the clustered results in **Figure 2**. As shown in **Figure 4B**, 10 different conserved motifs were obtained in HcNACs using MEME online software, and 53 of 113 HcNAC proteins contained 8 common motifs except for motifs 3 and 9, suggesting their important biological functions to be determined. However, motif 9 was exclusive to the NAC-F subgroup (HcNAC2, HcNAC27, HcNAC29, HcNAC60, HcNAC78, HcNAC80, and HcNAC81), indicating the specific functions of different subgroups might be owing to specific motifs. Intron/exon compositions were analyzed to gain the evolution information of *HcNAC* members (**Figure 4C**). Of the 113 *HcNACs*, more than 50% contained three code sequences. The number of intron regions was mainly 2 and varied from 1 to 12, proving that significant variation existed in the gene structure of *HcNAC* genes. Additionally, genes within each subgroup were similar in intron–exon structures. These results suggested that HcNACs clustered in the same subgroup shared similar conserved motifs and exon–intron organizations.

### 3.3 Gene duplication and collinearity analysis of *HcNACs* in *H. citrina*, *A. thaliana*, and *O. sativa*

**Figure 5:**
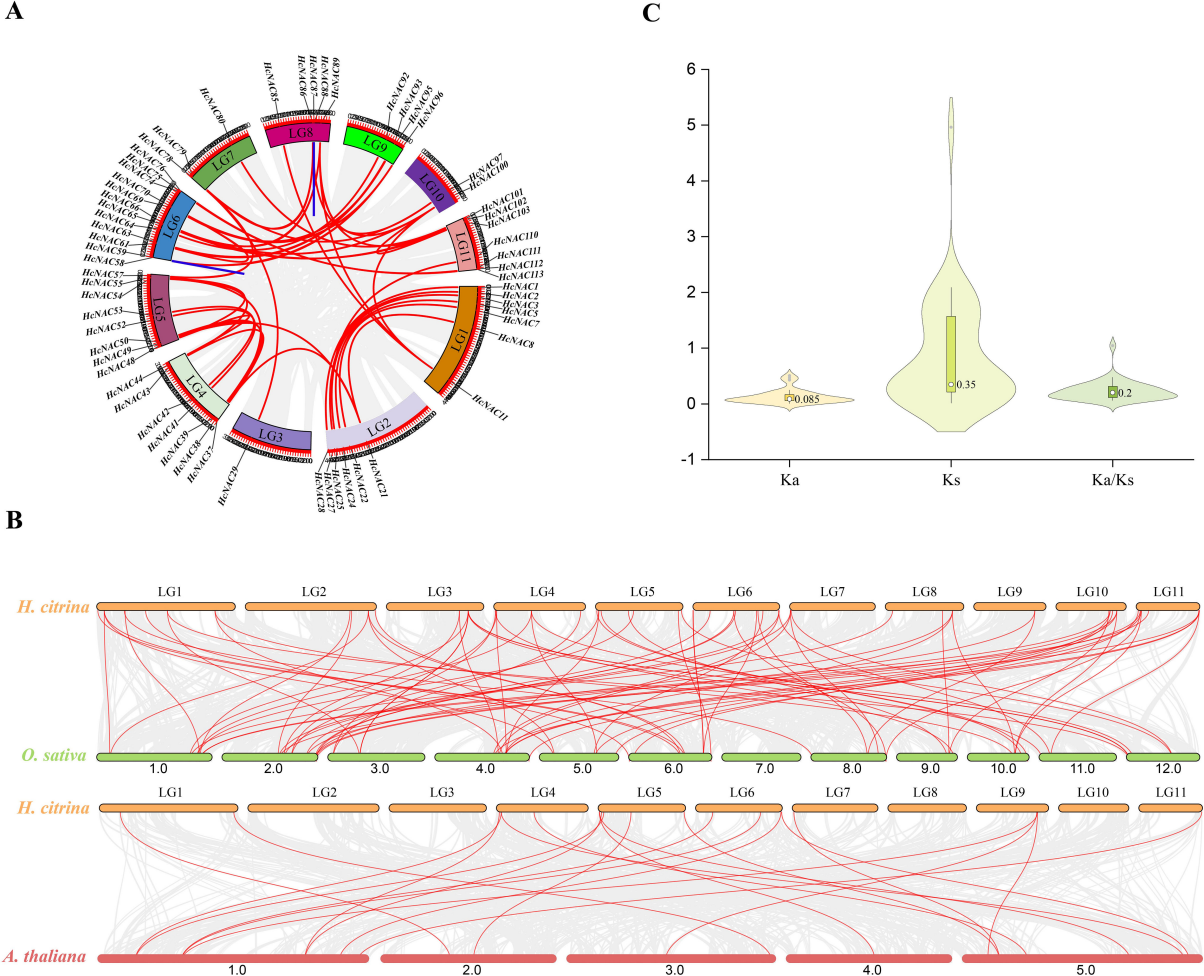
Collinearity analysis of *NAC* gene and Ka/Ks value in *H*. *citrina*. **(A)** Schematic representations for distribution and inter-chromosomal relationships of *NACs* in the *H*. *citrina* genome. The duplicated gene pairs were displayed in red lines, while the tandemly duplicated gene pairs were displayed in blue. **(B)** Synteny analysis between the *NAC* genes of *H*. *citrina* and two other representative plants including *O. sativa* and *A*. *thaliana*. **(C)** The Ka, Ks, and Ka/Ks values of *NAC* genes in *H*. *citrina*.

As effective methods for inferring the evolutionary history of species genome, intra- and inter-species collinear analysis are indispensable to address the study of *NAC* family expansions (Qiao et al., 2019). Through the annotation and intragenomic synteny analysis of *HcNAC* genes, 78 syntenic pairs were identified, among which two pairs of tandemly duplicated genes were detected (**Figure 5A**). It can be seen that tandem duplication and segment duplication might be major driving forces that form the expansion of the *HcNAC* gene family. Comparative syntenic maps of night lily with one monocot (*O. sativa*) and one dicot (*A. thaliana*) were constructed (**Figure 5B**). As a result, a comparison between night lily and *O. sativa* showed that 85 orthologous gene pairs were presented, whereas 21 *HcNAC* genes formed collinearity pairs with *NAC* genes from *A. thaliana.* The number of collinear *NAC* genes was higher in *O. sativa* than in *A. thaliana*, reflecting the closer relationship between night lily and monocots. The Ka, Ks, and Ka/Ks ratio, measurements of the protein conservation, were used to determine whether selective pressures occurred on genes encoding the HcNAC proteins (**Figure 5C** and **Supplementary Table S4**). The Ka/Ks value of only one *HcNAC* gene pair was greater than 1, which signified strong positive selection. The remaining genes were subjected to purification selection considering Ka/Ks ratios less than 0.5. The results revealed that the *HcNAC* gene family was mainly affected by purifying selection in evolutionary selection.

### 3.4 Functional Gene Ontology, and Kyoto Encyclopedia of Genes and Genomes enrichment of HcNACs

**Figure 6:**
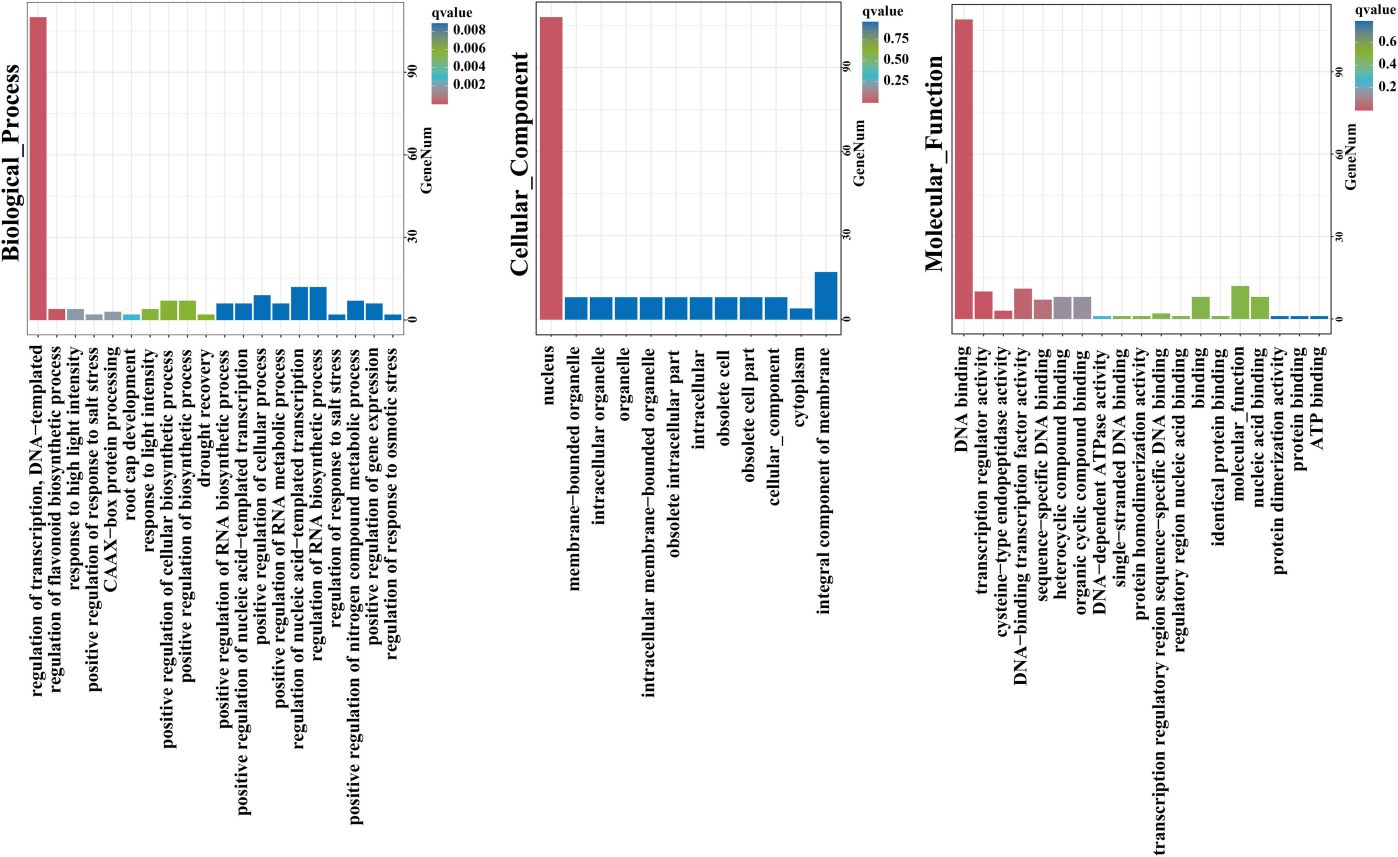
GO enrichment analysis of HcNACs in night lily.

Gene Ontology (GO) analysis of 113 HcNACs were subsequently performed to exposit biological processes, molecular functions, and cellular components (**Figure 6**). The enrichment results showed that the cellular component was mainly the nucleus, accounting for 108 of 113. HcNAC34, HcNAC42, HcNAC87, HcNAC88, and HcNAC94 were not enriched. For the category of biological process, all other NAC proteins except HcNAC33, HcNAC34, and HcNAC42 of night lily were enriched in the regulation of transcription. Furthermore, some HcNAC proteins were enriched in the regulation of nucleic acid–templated transcription, regulation of RNA biosynthetic process, and response to abiotic stresses. The main enriched function was functional DNA binding, accounting for 109 of 113 total functions, and either HcNAC34, HcNAC42, HcNAC80, or HcNAC106 were enriched. The next most enriched function was DNA-binding TF activity (total of 11 of 113). Kyoto Encyclopedia of Genes and Genomes (KEGG) enrichment analysis showed that most HcNACs except HcNAC106 were not involved in any pathway (**Supplementary Table S5**). The HcNAC protein GO enrichment information was provided in **Supplementary Table S5**.

### 3.5 Expression patterns of *HcNAC* genes in different tissues and *cis*-regulatory element analysis in promoter regions

**Figure 7:**
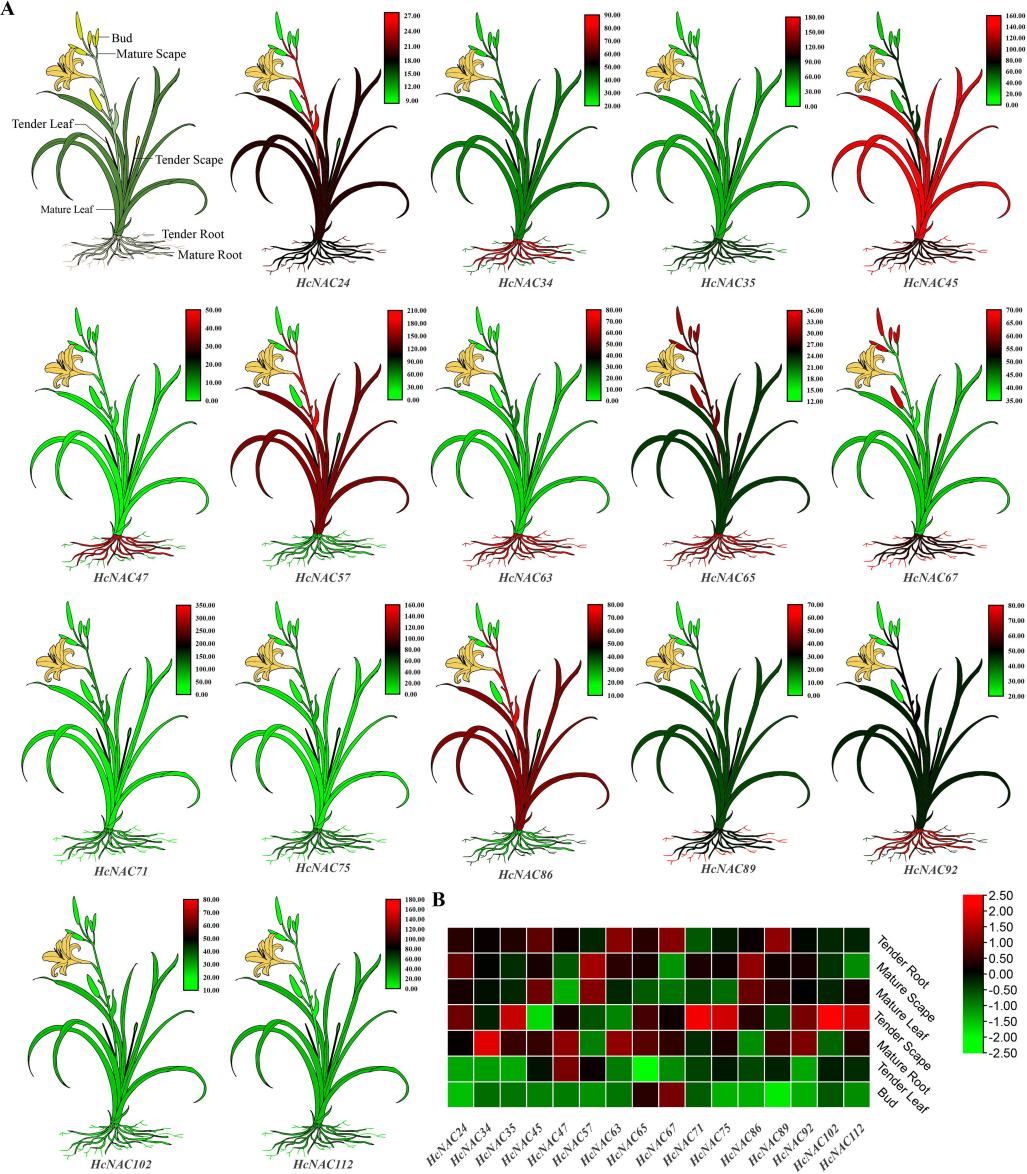
Diagram and expression heat maps of the selected *HcNAC* genes in different tissues. **(A)** Diagram for various tissues of night lily. Annotation of different tissues was shown in the first figure. The remaining 16 plants represented cartoon heat maps with red representing high gene expression and green representing low expression by RT-qPCR. **(B)** Expression heat map of 16 *HcNACs* in different tissues based on RNA-Seq data.

Seven different tissues, including bud, tender leaf, mature leaf, tender root, mature root, tender scape, and mature scape were used to check the tissue-specific expressions of the identified 113 *HcNACs.* RNA-Seq data were shown in **Supplementary Table S6**. Based on the analysis, the expression of *HcNAC* genes was detected in selected tissues, whereas their expression levels varied considerably (**Supplementary Figure S1**). Except for eight of the *HcNACs* not having expression data, the expressions of remaining genes in roots were relatively higher than the other tissues. Furthermore, 21 of 105 genes showed high transcript abundances in all tissues. To validate the RNA-Seq result, 16 *HcNACs* were selected for RT-qPCR analysis (**Figure 7A**). The expression levels were consistent with those from RNA-Seq data (**Figure 7B**). The results described herein represented that *HcNACs* might perform distinct functions through different tissues and be worth further studies.

**Figure 8:**
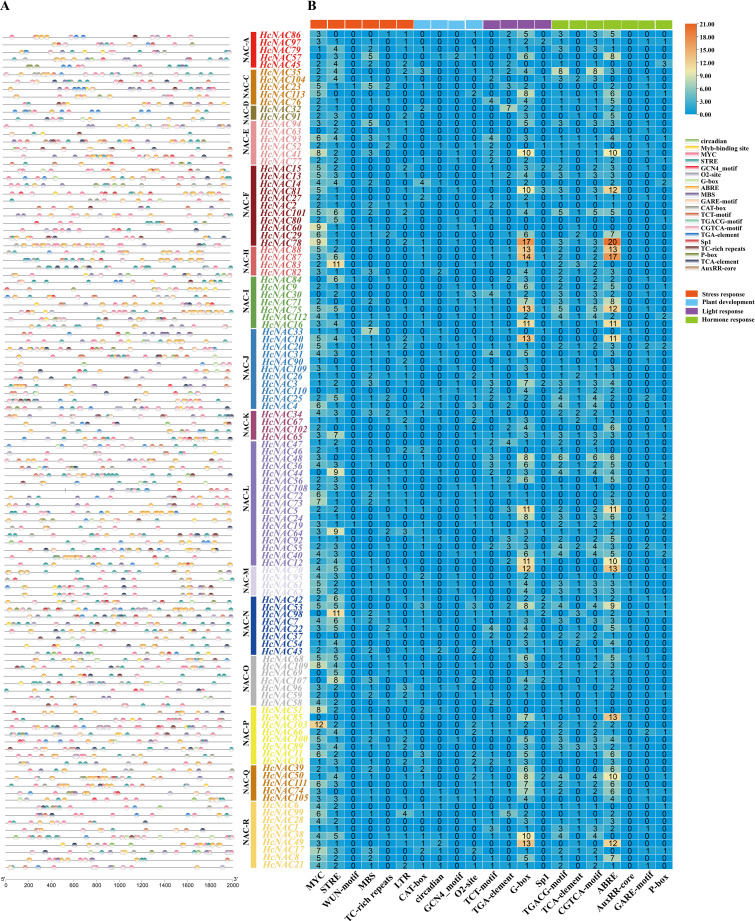
Distributions and numbers of *cis*-acting elements of the *HcNAC* genes. **(A)** The positions of diverse *cis*-acting elements in the promoter region. *HcNACs* belonging to different subgroups were denoted with different colors. **(B)** The corresponding number of *cis*-acting elements in the promoter region of each *HcNAC* gene.

To elucidate the potential transcriptional regulation of *HcNAC* genes, *cis*-acting elements of the upstream promoter regions (2,000 base pairs) were classified and analyzed (**Figure 8** and **Supplementary Table S8**). A total of 115 abundant regulatory elements were detected via the online PlantCARE database, *HcNAC111* hosted 295 cis-elements whose number was the largest (**Supplementary Table S7**). We selected 21 typical responsive cis-elements to further unveil transcriptional regulation of *HcNAC* genes (**Figure 7**; **Supplementary Table S8**). All *HcNAC* genes had regulatory elements linked to stress response, involving defense and stress, wound, drought, and low-temperature responsive elements. In addition, elements of CAT-box, circadian, GCN4_motif, and O2-site were identified as key regulars of plant development. Light-response covered TCT-motif, TGA-elements, G-box, and SpI elements. Hormone-related elements were associated with auxin, abscisic acid, gibberellin, salicylic acid, and MeJA, respectively, CGTCA-motif, ABRE, AuxRR-core, GARE-motif, and P-box. Our analysis revealed the presence of different types of *cis*-acting elements in the *HcNAC* gene family, which potentially regulate plant growth and development, and in response to hormone and abiotic stress.

### 3.6 Expression profiles of *HcNAC* genes under drought and salinity stresses

**Figure 9:**
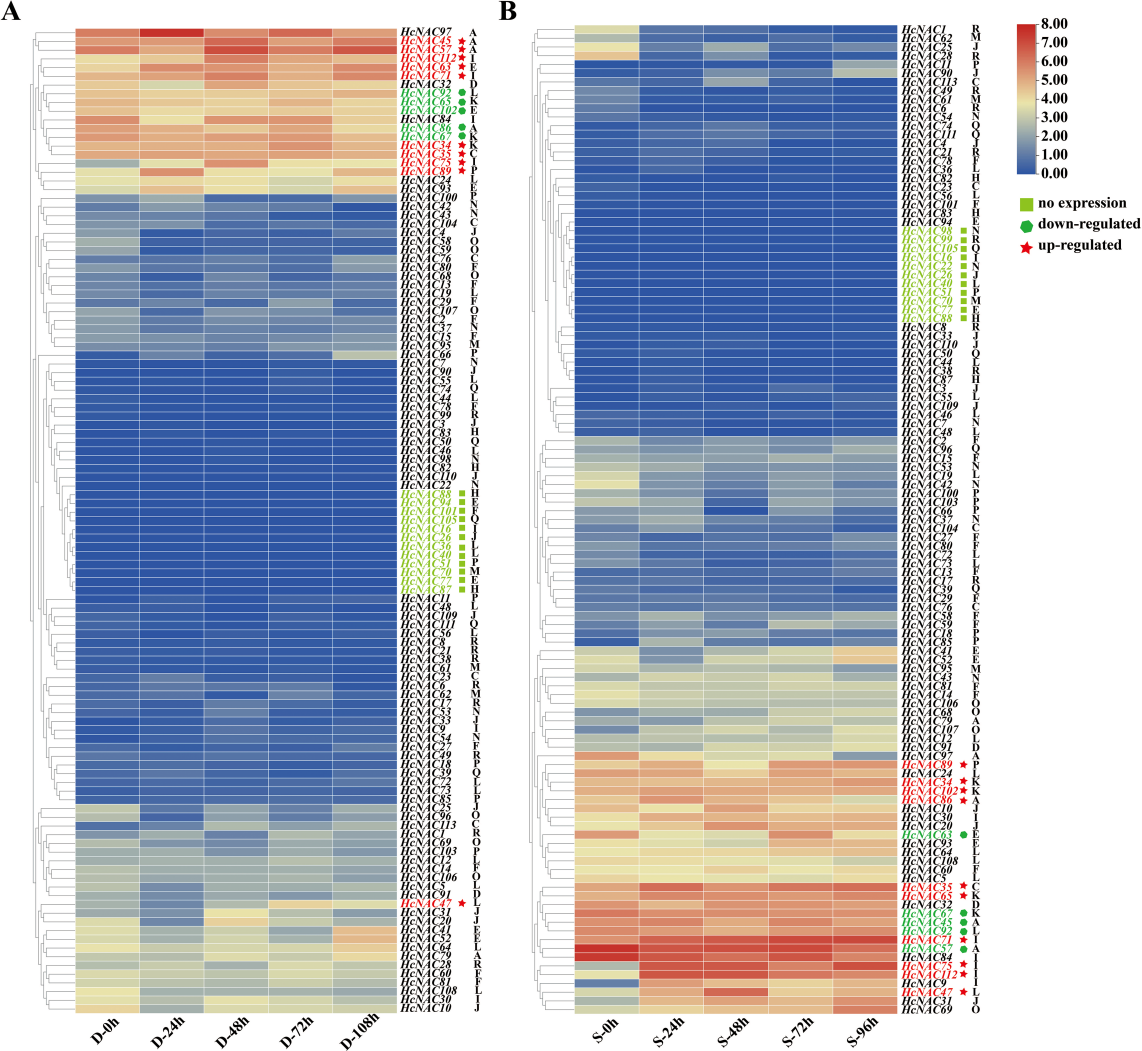
Expression analysis of Hc*NACs* under abiotic stresses based on RNA-Seq data. **(A, B)** Expression heat maps of *HcNACs* under drought **(A)** and salinity **(B)** stresses. Hierarchical clustering was used in the data analysis. A, C–F, and H–R represented 16 subgroups based on phylogenetic analysis. Red and blue colors indicated high- and low-expression levels calculated with log_2_FPKM values. Different types of genes in expression were marked with different shapes and colors.

To better illustrate the possible roles of *HcNAC*s in abiotic stress, we collected night lily roots to carry out RNA-Seq sequencing to identify the expression patterns of *HcNAC* genes after drought (PEG) treatment for 0–108 h and salt (NaCl) treatment for 0–96 h. The expressions of night lily *HcNACs* under drought stress were shown in **Supplementary Tables S9**, **S10** under salinity stress. In 16 subgroups, a total of 12 *HcNACs* of night lily were detected with no expressions when suffering from drought while 11 were under salinity stress (**Figures 9A, B**). As the treatment time increased from 0 to 108 h under drought stress and 96 h under salinity stress, 10 *HcNAC* genes were upregulated and 5 genes were downregulated, among which *HcNAC*57 and *HcNAC*86 belonging to the NAC-A subgroup exhibited the opposite trends under two stresses. The results from RNA-Seq data revealed that the *HcNAC* genes were likely to function distinguishably in response to different abiotic stresses.

According to the analysis of *cis*-elements of upstream 2,000 bp sequence of night lily *NAC* genes (**Figure 8** and **Supplementary Table S8**), most of *HcNACs* in 16 subgroups contained six general stress response elements: MYC, stress response element (STRE), WUN-motif, MYB-binding sites (MBS), TC-rich repeat (*cis*-acting factor involved in defense and stress response), and low-temperature response motif (LTR). They accounted for 40%, 34%, 1%, 12%, 5%, and 8% of the total number of cis-elements identified in abiotic and biotic stress categories, respectively. In 16 genes which showed high expression in different tissues and abiotic stresses, the number of stress response elements ranged from 2 to 14. Among them, seven *HcNAC* genes (*HcNAC*34, *HcNAC*35, *HcNAC*45, *HcNAC*57, *HcNAC*65, *HcNAC*75, and *HcNAC*89) contain more stress response elements than other genes. *HcNAC63* in the NAC-E subgroup did not contain MYC response element involved in abiotic stress response. Three genes (*HcNAC71*, *HcNAC86*, and *HcNAC102*) did not contain STRE response element. It was speculated that *HcNACs* might be involved in diverse abiotic stress responses and regulatory pathways.

**Figure 10:**
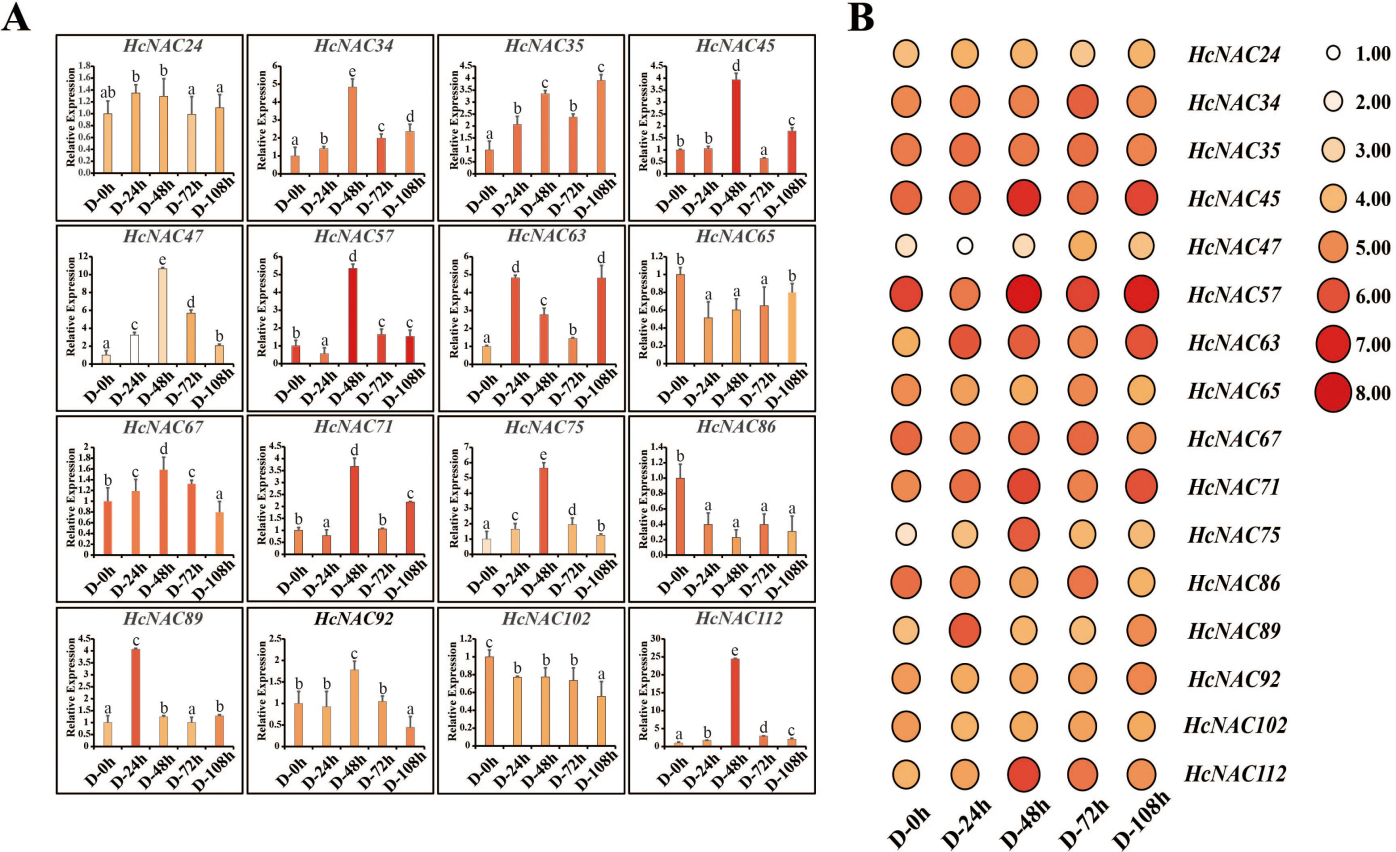
The expression of 16 *HcNAC* genes under drought stress (20% PEG) for 0–108 h in roots. **(A)** Expression levels of the *HcNAC*s after drought treatment by RT-qPCR. The lowercase letters indicated statistically significant differences (*p* < 0.05). **(B)** Expression patterns of the *HcNAC*s after drought treatment based on transcriptome data.

Several *NAC* genes have been reported to regulate the growth and development of plants against abiotic stress (Huang et al., 2013). To gain insights into the putative functions of *HcNAC* genes in growth and development, the transcription levels of 16 genes, which showed significant expression disparities under PEG treatment, were scrutinized by RT-qPCR (**Figure 10A**). *HcNAC65*, *HcNAC86*, and *HcNAC102* were downregulated. On the contrary, seven *HcNAC* genes (*HcNAC34*, *HcNAC35*, *HcNAC47*, *HcNAC63*, *HcNAC75*, *HcNAC89*, and *HcNAC112*) showed an increasing trend with time extension after treatment. Furthermore, *HcNAC35* showed the most significant upregulation by drought stress. Therefore, *HcNAC35* was considered a candidate gene for abiotic stress responses, and further studies of the gene expression during PEG treatment were needed. The results hinted at the consistency of RT-qPCR and transcriptome results under drought stress (**Figures 10A, B**), which provided a crucial reference for functional gene selection in the *NAC* gene family of *H. citrina*.

**Figure 11:**
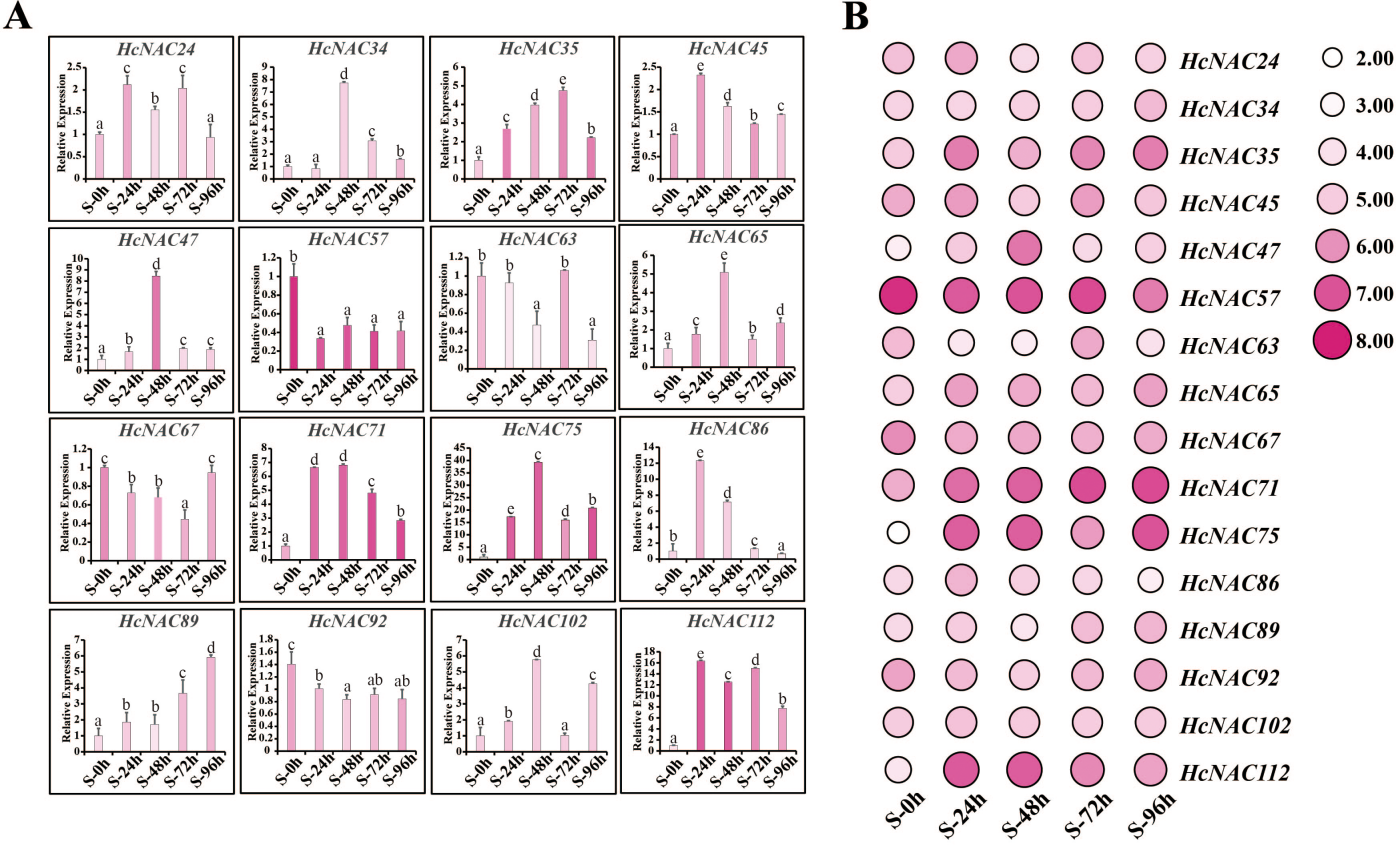
Expression levels of the selected *HcNAC* genes under salinity stress (250 mM NaCl) for 0–96 h in roots. **(A)** Relative expression levels of the *HcNAC*s after salt treatment by RT-qPCR. The lowercase letters indicated statistically significant differences (p < 0.05). **(B)** Expression patterns of the *HcNAC*s after salt treatment based on RNA-Seq data.

For salinity stress, we also performed RT-qPCR experiments to confirm the data accuracy of the selected 16 genes. As observed in **Figure 11A**, the expression of *HcNAC57* displayed a decrease of twofold to threefold obviously while *HcNAC67* was downregulated slightly, being suppressed at all processing times. In contract, eight genes (*HcNAC35*, *HcNAC47*, *HcNAC65*, *HcNAC71*, *HcNAC75*, *HcNAC89*, *HcNAC102*, and *HcNAC112*) exhibited upregulation after NaCl treatment. Notably, the expression of *HcNAC35* and *HcNAC71* were significantly increased by threefold to sevenfold at 24–96 h of treatment in response to salinity stress. Therefore, the consistency was equally manifested between RNA-Seq and qPCR results under salinity stress (**Figures 11A, B**), indicating that the sequencing data were highly reproducible. Overall, the expression patterns of *HcNAC* genes under different stress conditions proved that down- or up-expressed genes might engage in various stress responses.

### 3.7 Subcellular localization of *HcNAC35* and *HcNAC71*

**Figure 12:**
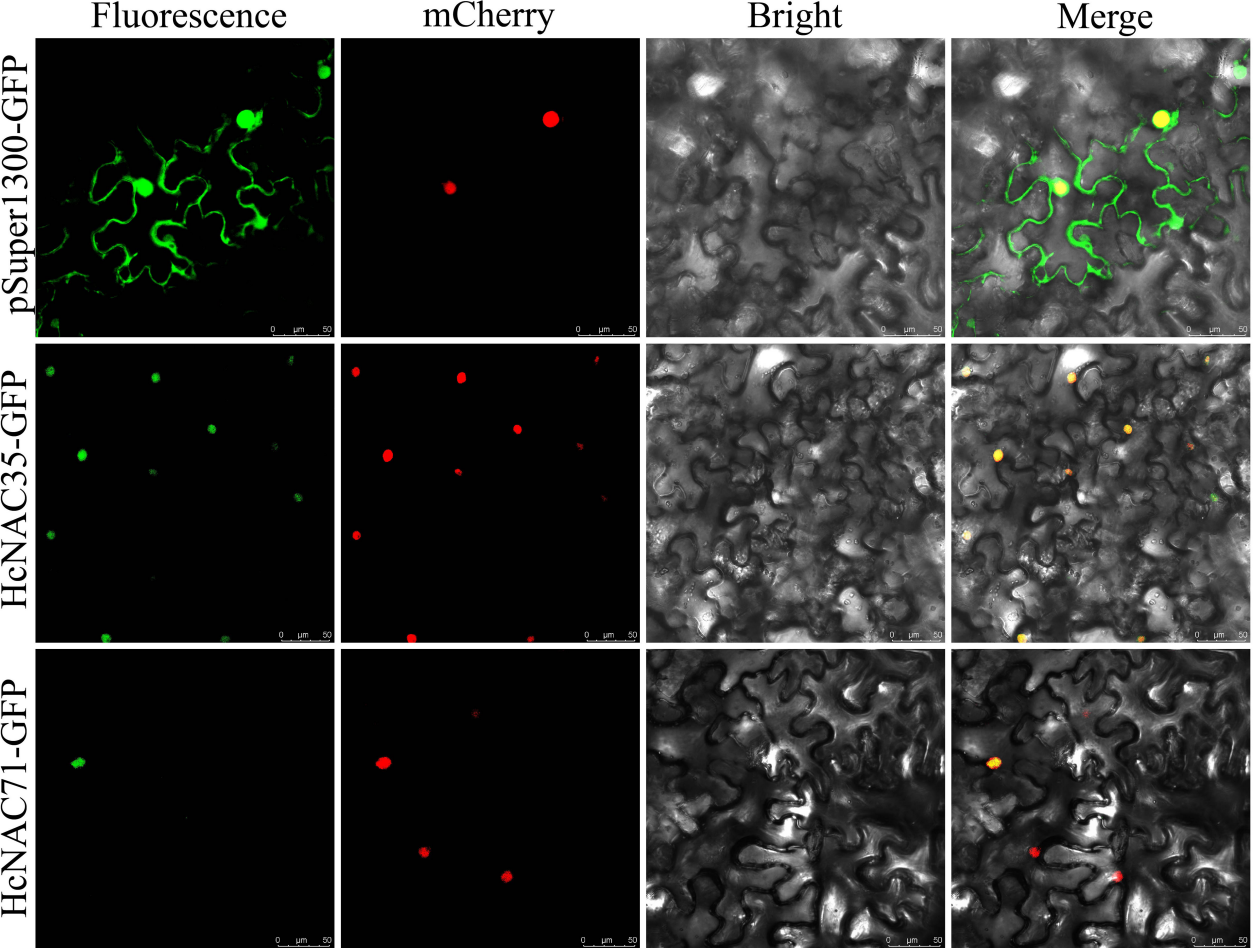
Subcellular localization of *HcNAC35* and *HcNAC71*. Empty pSuper1300:GFP was used as the negative control. The second panel mCherry represented a positive marker for the nucleus. The merged image (rightmost panel) indicated the fusion of GFP (green fluorescence), mCherry (red fluorescence), and bright field. Bar = 50 μm.

Subcellular localization is known to be used to predict protein function. As one of the gene families classified into the TFs, most *HcNAC* genes in addition to *HcNAC35* and *HcNAC71* were predicted to locate in the nucleus (**Supplementary Table S2**). To test the accuracy of predicted results, we selected *HcNAC35* and *HcNAC71*, which exhibited distinct patterns in expression under drought and salt treatment, to construct HcNACs-GFP fusion proteins. As shown in **Figure 12**, the results of the empty vector showed that strong fluorescence signals could be detected in the nucleus, cytoplasm, and cell membrane when pSuper1300-GFP was transformed. The signal location of HcNAC35-GFP and HcNAC71-GFP was only within the nucleus, which was the same as the predictions. It is speculated that *HcNAC35* and *HcNAC71* may play a role in the nuclear regulation as typical TFs, and the deep specific functions remain to be uncovered in future studies.

### 3.8 Overexpression of *HcNAC35* improved drought and salt tolerance in watermelon

**Figure 13:**
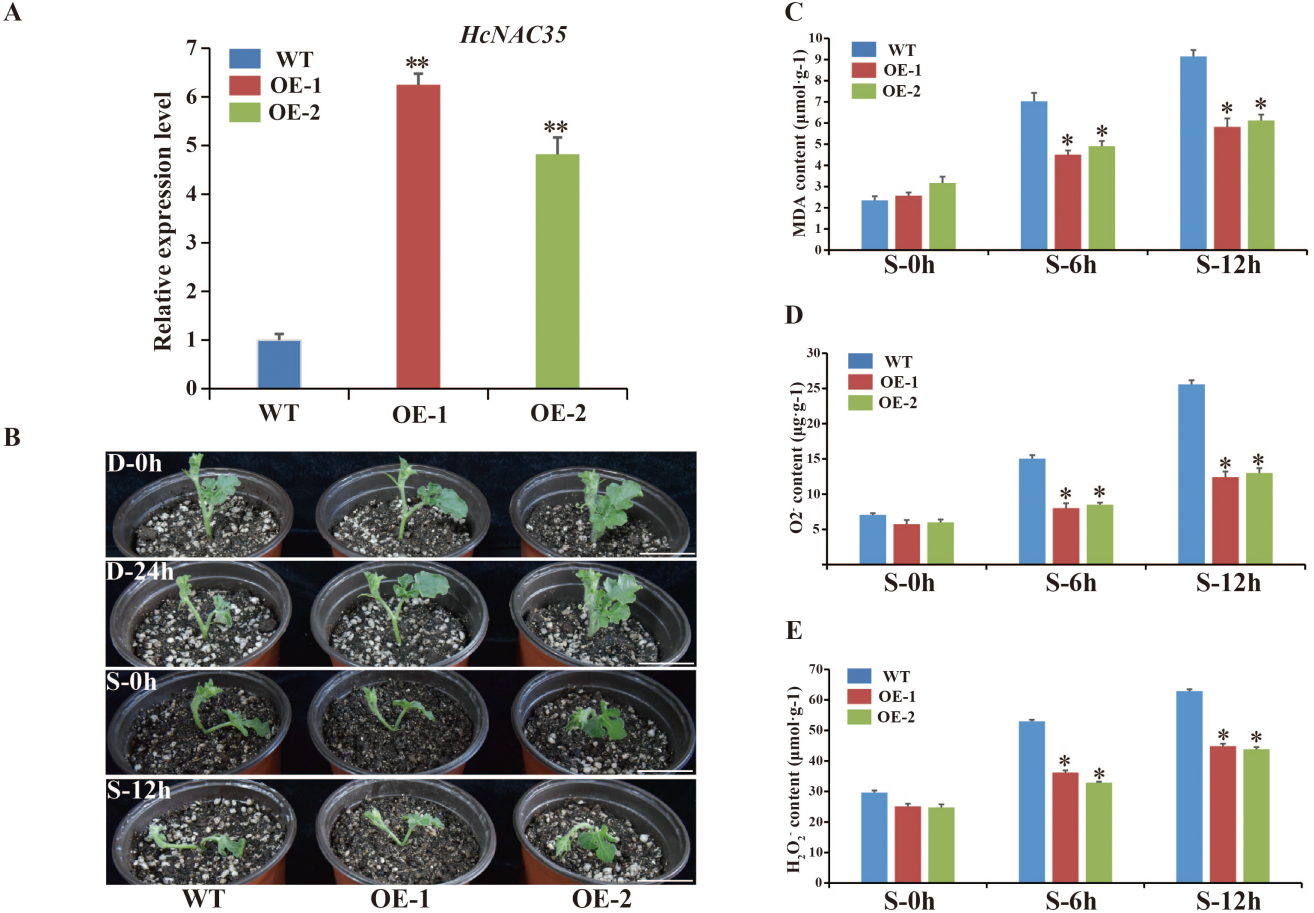
Performance and responses of *HcNAC*-OE plants of watermelon to salt and drought stresses. **(A)** Relative expression of *HcNAC35* among WT and OE plants by RT-qPCR. **(B)** The phenotypes of the WT and *HcNAC*-OE plants under abiotic stress conditions (D, drought; S, salt). Bar = 4 cm. **(C–E)** MDA **(C)**, O2^−^ **(D)**, and H_2_O_2_ **(E)** contents in leaves from plants under normal and salt stress. The significant variations were marked by asterisk(s) (*p < 0.05, **p < 0.01).

Previous experiments have implied that *HcNAC35* exhibited significant upregulation under drought and salt treatments. To investigate the biological function of *HcNAC35* in watermelon abiotic stress responses, we generated *HcNAC35*-OE plants in the TC watermelon genetic background. Based on expression levels of *HcNAC35*, two independent transgenic lines were chosen for further analysis. The RT-qPCR results showed that the transcript abundance of *HcNAC35* in the OE-1 and OE-2 plants was about 4.82-fold and 6.25-fold higher than WT plants, respectively (**Figure 13A**).

To further assess whether alteration of *HcNAC35* expression affects drought and salinity stress tolerance, we treated 4-week-old *HcNAC35-*OE plants and WT seedlings with 25% PEG and 250 mM NaCl, respectively (**Figure 13B**). The obvious wilting was observed in the shoot tips (STs) and leaves of WT seedlings after 12 h under 250 mM NaCl treatment, while STs and leaves of OE seedlings showed little damage. Additionally, visible damage was also caused in WT seedlings when 0–24 h under 25% PEG treatment. The phenotype observation indicated that *HcNAC35* might play a critical role in response to abiotic stresses, especially salinity stress.

As indicators of membrane peroxidation, contents of MDA were measured to further evaluate the damage degree under salinity stress. MDA levels increased in all treated plants, but 30% and 38% lower in the OE plants than those of WT plants when 0–12 h after salt treatment (**Figure 13C**), indicating that the overexpression of *HcNAC35* contributed to decreased damage under salinity stress. The levels of superoxide radicals (O_2_^−^) were determined to explore the change of sensitivity caused by altered redox status in OE plants under salinity stress. *HcNAC35*-overexpressing plants showed lower O_2_^-^ and H_2_O_2_ contents during salinity stress than the TC plants (**Figures 13D, E**). These results indicated that overexpression of *HcNAC35* increased the tolerances of watermelon to salt and drought stresses to different degrees.

## 4 Discussion

With members further supplemented by transcriptome and genome analysis, the NAC family has become one of the largest families of plant-special transcriptional regulators. As important switches to accurately regulate gene expression, NACs play pivotal roles in regulating plant development and various physiological processes in response to abiotic stress (Kumar et al., 2021; Singh et al., 2021). NAM, ATAF1/2, and CUC2 constitute the NAC acronym, and they were initially discovered to hold a common conserved NAC domain (Souer et al., 1996; Aida et al., 1997; Ooka et al., 2003). The NAC TF family, identified in a variety of species, remains undescribed in night lily so far (Ooka et al., 2003; Nuruzzaman et al., 2010; Su et al., 2013; Wang N. et al., 2013; Le et al., 2011; Yan et al., 2017; Li et al., 2018; Gong et al., 2019; Jin et al., 2020; Li et al., 2020; Nie et al., 2020; Zong et al., 2020; Li et al., 2021; Meng L. et al., 2022). Based on previously reported genome sequences, 113 *NAC* gene family members were identified and randomly distributed on 11 chromosomes (**Figure 1**), similar to the corresponding families of other angiosperms in classification standard and number (Huang et al., 2013; Liu et al., 2023). This may reflect the relative stability of the *HcNAC* family evolution process. A total of 18 subgroups were classified among *H. citrina*, *O. sativa*, *A. thaliana*, and *C. lanatus.* However, the number of four species in individual subgroups was discrepant (**Figure 2**), indicating that although different NAC family proteins originate from the same ancestor, they evolve distinguishably among species.

Extensive variations existed in protein length, predicted molecular weight, and isoelectric point (**Supplementary Table S1**). In contrast, of identified 113 *HcNAC* genes, NAM domain was detected in 112 NAC proteins, and more than 50% contained two CDS regions (**Figures 3**, **4**), indicating relative conservation of gene structures and functions of the *NAC* family. The diversity of gene structure occurs in the evolutionary process of numerous gene families, which is valuable for excavating potential new functions to adjust to environmental changes (Liu et al., 2023). In general, the members belonging to the same phylogenetic group possess a high degree of similarity in gene structure and conserved motif (**Figure 4**), suggesting they have a closer phylogenetic relationship (Zhang et al., 2014). Motifs made up of short sequences are involved in important biological processes. The conserved motif analysis of *HcNACs* showed high coverage to the conserved protein region. Moreover, members of different subfamilies may contain non-identical motifs, but hold the DNA binding domain, consistent with those reported in *Liriodendron* (Liu et al., 2023).

Currently, duplication events of *NAC* genes including segmental and tandem duplications have been widely reported in different plant species (Li et al., 2020; Shan et al., 2020). In our study, 2 tandem and 78 segmental duplication events were screened out in the *HcNAC* genes (**Figure 5A**), revealing that segmental duplication might be the main force for forming and expanding the *NAC* gene families (Song H.Y. et al., 2022). The collinear relationships of NACs between night lily and monocotyledon NAC family were found to be greater, and less with dicotyledon (**Figure 5B**), which may be associated with the classification of monocot and dicot plants produced by angiosperms during long-term natural selection and evolution (Xiong et al., 2024). In particular, the high similarity between homologous gene pairs was detected in constructed gene structures and predicted protein properties. This result suggests that duplicate genes derived from the progenitors can evolve separately simultaneously and show few changes (Wang et al., 2010). We calculated the Ka/Ks value for selective pressure analysis, finding purifying selection as a primary force in the evolutionary process of *NAC* genes in night lily (**Figure 5C** and **Supplementary Table S4**), concluding that they might retain primitive functions from their ancestry (Li et al., 2016).

To be public knowledge, gene functional enrichment analysis is a requisite method to elaborate the biological processes and signaling pathway. In this study, we found that members of the identified *HcNAC* gene family were widely involved in abiotic stress responses and regulation of transcription (**Figure 6** and **Supplementary Table S5**), indicating their potentially significant roles in dealing with abiotic stress. The promoter structures and their regulatory pathways are tightly associated with many plant traits (Wu et al., 2018). Multiple regulatory elements with central physiological functions in the *HcNAC* promoters implied that *HcNACs* might respond to various internal factors (growth and development) and external factors (abiotic stresses) (**Figure 8** and **Supplementary Table S8**). Our results revealed that *HcNAC35* contained eight stress response elements (two MYC, four STRE, and two LTR), similar to seven stress response elements (three MYC, one STRE, two LTR, and one MBS) of the homolog *At3g10500* (*ANACO53)* in *Arabidopsis* (Olivier et al., 2016). Taken together, it is noteworthy that these genes might play conserved functions in stress responses across species (Cao et al., 2024).

The analysis of expression patterns can be one effective way to explore the functions and evolutionary relationships of gene families (Wang Y. et al., 2013). Our study implied that most of the *HcNAC* genes displayed tissue-specific expression through RNA-Seq and RT-qPCR analysis (**Figure 7** and **Supplementary Figure S1**); furthermore, the relatively higher expressions were preferentially concentrated on roots. These results provided useful clues for understanding gene functions concerning specific physiological processes. Several researches have been conducted to find out the roles of NAC TFs in coping with diverse stresses, such as salinity, drought, and flooding (Singh et al., 2021; Puranik et al., 2012; Yuan et al., 2020). Likewise, RNA-Seq and RT-qPCR were also combined to check the expression patterns of *HcNAC* genes under two abiotic stresses (**Figure 9** and **Supplementary Tables S10**, **S11**). The expressions of most *HcNAC* genes changed dramatically at 48 h after drought and salinity stresses, which might be due to the sudden increase of osmotic pressure, leading to the reduction of enzymatic activity in the plant. We speculated that the defense system of the plant has been fully activated after stress treatment for about 72 h, and gene expressions related to stress response in addition to *HcNACs* were enhanced through signal transmission. In particular, the expression levels of *HcNAC35* and *HcNAC71* holding the NAM domain fluctuated significantly (**Figures 10**, **11**), implying that they might perform remarkable functions as TFs in response to diverse abiotic stresses. Furthermore, our investigation shows most NAC TFs including NAC35 and NAC71 in night lily have been predicted to locate in the nucleus, consistent with our experimental results (**Figure 12**). Overexpression of *HcNAC35* in watermelon further indicated the strong resistance to abiotic stresses (**Figure 13**). However, whether notable developmental anomalies are caused remains to be continuously explored based on other phenotypes of *HcNAC35-*OE plants in subsequent growth and development. Thus, further investigations are needed to clarify the cellular mechanisms of the *HcNAC35* regulatory gene in watermelon.

## 5 Conclusions

In this study, a genome-wide characterization of NAC TFs was comprehensively performed in night lily. We identified 113 *HcNACs* encoding NAC TFs, which were unevenly distributed across 11 chromosomes. Based on the evolutionary relationship, HcNACs could be divided into 18 distinct subgroups. The identified HcNAC proteins have a closer evolutionary relationship with *O. sativa* proteins, suggesting their higher similarities with NACs of monocots in the natural selection and evolution. The identified *cis*-acting elements were predicted to regulate different biological processes, reflecting the diversification of *HcNAC* genes in function. Our RNA-Seq data and RT-qPCR results identically showed that *HcNAC* genes expressed specifically and distinctly in different tissues. Moreover, HcNACs especially HcNAC35 and HcNAC71 might be involved in response to environmental stresses, including drought and salinity. Overexpression of *HcNAC35* in watermelon enhanced abiotic stress tolerances, especially salinity, and might affect leaf development. These results help to elucidate the response of *HcNAC35* to abiotic stress and set the stage for further functional research on *NAC35* in perennial crops. However, whether these NAC TFs perform regulatory roles in the growth and development of night lily remains to be further confirmed in future studies.

## Data Availability

The data presented in the study are deposited in the NCBI repository, accession numbers PRJNA1154840, PRJNA1154841, and PRJNA1154842.
