# Supplementary material for: Genome-wide identification of the NAC family in Hemerocallis citrina and functional analysis of HcNAC35 in response to abiotic stress in watermelon
Source: Front Plant Sci. 2024 Oct 14;15:1474589. doi: 10.3389/fpls.2024.1474589 (PMC11513300; doi:10.3389/fpls.2024.1474589)
Supplement: Supplementary file 12 [file Table12.docx]

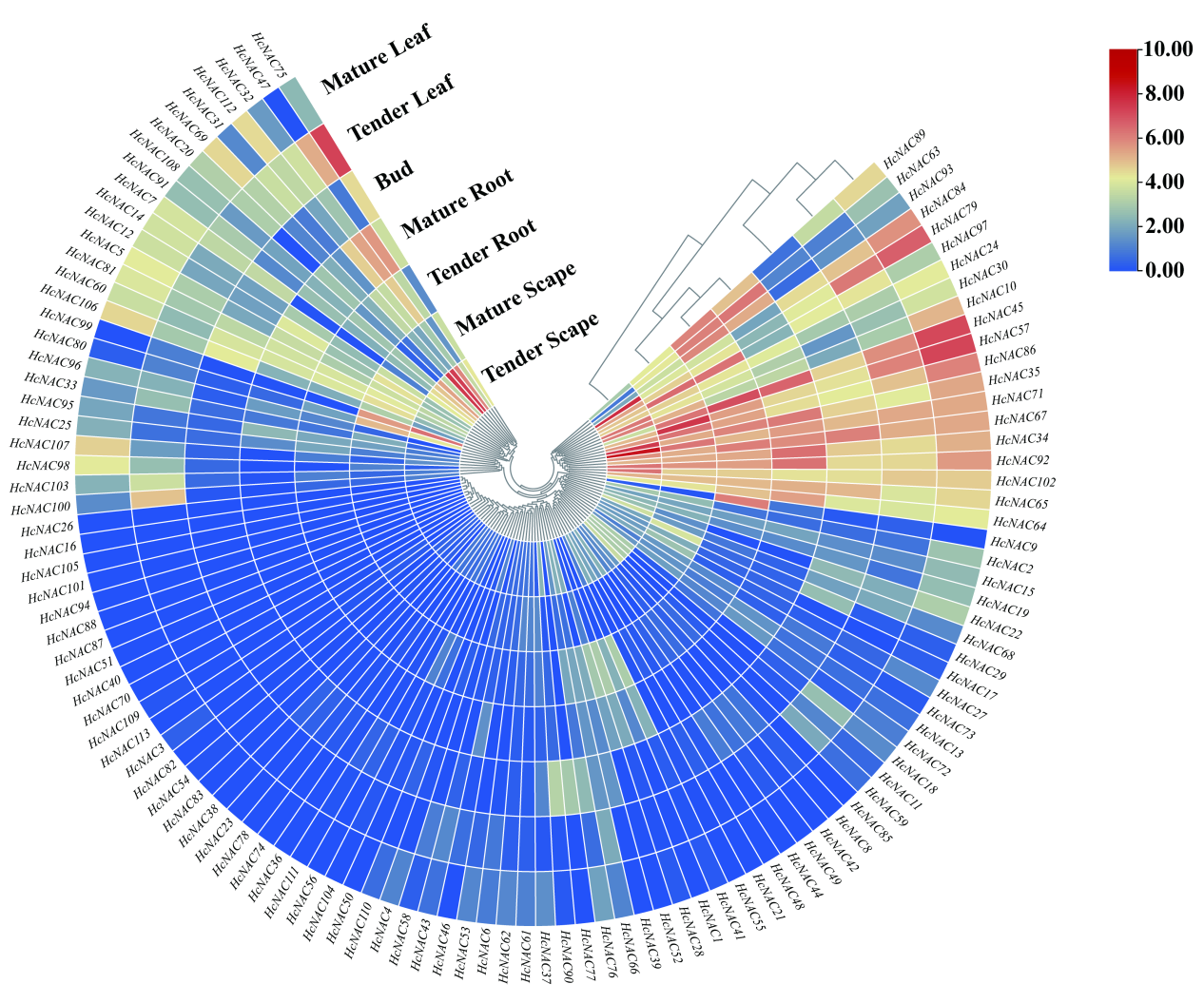


**Supplementary Figure 1.** Expression analysis of *NACs* in *H. citrina* in different tissues based on RNA-seq data. Red and blue colors indicated high and low expression levels calculated with FPKM values.
